# Central auditory processing: behavioral and electrophysiological assessment of children and adolescents diagnosed with stroke

**DOI:** 10.1016/j.bjorl.2019.10.010

**Published:** 2019-12-09

**Authors:** Amanda Zanatta Berticelli, Claudine Devicari Bueno, Vanessa Onzi Rocha, Josiane Ranzan, Rudimar dos Santos Riesgo, Pricila Sleifer

**Affiliations:** a Universidade Federal do Rio Grande do Sul, Programa de Pós-Graduação em Saúde da Criança e do Adolescente, Porto Alegre, RS, Brazil; b Pontifícia Universidade Católica do Rio Grande do Sul, Programa de Pós-Graduação em Medicina: Neurociências, Porto Alegre, RS, Brazil; c Hospital de Clínicas de Porto Alegre (HCPA), Porto Alegre, RS, Brazil; d Universidade Federal do Rio Grande do Sul, Departamento de Saúde e Comunicação Humana, Porto Alegre, RS, Brazil

**Keywords:** Stroke, Evoked potentials, auditory, Auditory perceptual disorders, Auditory diseases, central, Child

## Abstract

**Introduction:**

Central auditory processing refers to the efficiency and effectiveness with which the central nervous system uses auditory information: it may be altered in neurological disorders and brain injuries, such as strokes. However, despite evidence of probable alterations in the pediatric population, functional abilities and post-stroke limitations are still not well documented in the literature.

**Objective:**

To analyze the findings of the electrophysiological and behavioral evaluations of central auditory processing of children and adolescents diagnosed with stroke from a reference outpatient clinic, as well as to investigate possible associations with the variables: type and location of the stroke and age group.

**Methods:**

The present study is characterized as comparative cross-sectional. The sample, for convenience, included individuals aged 7–18 years divided into two groups: study group, composed of individuals with a diagnosis of stroke, and control group, composed of individuals with typical development. The evaluation consisted of the following procedures: anamnesis, basic audiological evaluation, behavioral evaluation of the auditory processing disorder (dichotic digit test, dichotic consonant-vowel, synthetic sentence identification/pediatric speech intelligibility, gaps in noise, pitch pattern sequence, masking level difference), and electrophysiological evaluation (P300 and mismatch negativity).

**Results:**

Nineteen children and adolescents were included in the study group. The control group was composed of 19 children and adolescents with typical development. In the comparison between the groups, a worse performance is observed for the study group in all the evaluated tests, behavioral and electrophysiological. In the behavioral evaluation of central auditory processing, there was a statistical difference for all tests, except for masking level difference and dichotic digit test, binaural separation step on the left. In the electrophysiological evaluation, there was a statistical difference in the latency of mismatch negativity and P300. No associations were found between the behavioral and electrophysiological findings and the location of the stroke and age group variables.

**Conclusion:**

Children and adolescents diagnosed with stroke present a worse performance in the electrophysiological and behavioral evaluations of central auditory processing when compared to a control group.

## Introduction

Auditory information processed in the brain is complex information that integrates auditory stimuli and cognitive-linguistic operations simultaneously and sequentially. Central auditory processing (CAP) refers to the efficiency and effectiveness with which the central auditory nervous system (CANS) uses auditory information.^1^

CAP involves a complex range of structures and functions that require the integrity of the peripheral auditory system and maturation of the central auditory nervous system.^2^ Thus, all structures of the auditory system, from the outer ear to the auditory cortex, have to be intact so that auditory information can be detected, transmitted and interpreted.^3^, ^4^

CAP can be accessed through behavioral and electrophysiological measurements. Behavioral tests assess different auditory skills and aim to investigate how individuals pay attention to, organize, memorize and perceive details of verbal and nonverbal auditory information.^5^ Electrophysiological tests allow the measurement of neuroelectric activity throughout the auditory pathway, providing further information about the functioning of the CANS. Therefore, processing of auditory information can be observed in the time domain.^6^, ^7^

Auditory processing disorder (APD) may be due to delayed maturation of the central auditory pathways, neurological disorders and brain lesions.^8^ Therefore, considering neurological changes as a risk factor for changes in CAP, children with a diagnosis of stroke are part of a risk group.^1^

Stroke is defined as a sudden occlusion or rupture of cerebral veins or arteries, with interruption of blood flow to the brain, resulting in focal brain injury and clinical neurological deficits.^9^, ^10^ Recent studies have indicated an incidence rate ranging from 1.2–13 cases per 100,000 children per year.^1^, ^2^, ^3^, ^4^, ^5^, ^6^, ^7^, ^8^, ^9^, ^10^, ^11^, ^12^, ^13^, ^14^ In children, mechanisms underlying the pathophysiology of stroke are still poorly understood.^11^, ^15^, ^16^ In addition, the etiology, presentation, evolution and outcome stroke in children are different from those described in adults.^17^

In the pediatric population, functional skills and post-stroke limitations are still not well-documented in the literature. Previous studies have reported neurological alterations such as motor, linguistic and cognitive deficits.^18^, ^19^ Although they are hardly investigated in the pediatric population, there is evidence of impairments in hearing abilities of CAP in children diagnosed with stroke.^20^, ^21^

As there is evidence of possible effects of stroke on auditory skills of CAP, these effects have to be taken into account for evaluation of children, because of functional limitations that result from these impairments, especially in the school and the social environments.

Given the above-mentioned considerations, the present study aims to investigate the findings of behavioral and electrophysiological assessments of central auditory processing of children and adolescents diagnosed with stroke at a referral outpatient clinic. Another objective was to investigate possible associations with the variable types of stroke, the side of stroke lesion and age group.

## Methods

The present study is a comparative cross-sectional study, carried out according to guidelines for human research, according to Resolution nº 466/12 and approved by the Ethics Committee on Research with Human Beings of the Universidade Federal do Rio Grande do Sul (UFRGS) under protocol number 77900517.2.0000.5334.

The sample, for convenience, included individuals aged 7–18 years divided into two groups: Study Group (SG) composed of individuals with a diagnosis of stroke, who were followed at the Cerebrovascular Diseases Outpatient Clinic from Hospital de Clínicas de Porto Alegre’s Neuropediatric Unit, Porto Alegre, Brazil, and Control Group (CG), composed of children and adolescents with typical development. The CG was composed by children with good academic performance recruited from public and private schools of Porto Alegre, Rio Grande do Sul, Brazil, paired by age and sex. Participants under 12 years old were considered children and those aged 12–18 years old were considered adolescents. The data collection took place between May and December 2018.

For both groups, the inclusion criteria were: children and adolescents aged 7–18 years, presence of normal auditory thresholds^22^ and type A tympanogram curve.^23^ In addition, the presence of acoustic reflex activity bilaterally, absence of otological and audiological complaints, or complaints of learning difficulties at school were taken into consideration for the control group. For both groups, the exclusion criteria were presence of neurological sequelae that prevented audiological assessments.

All the participants underwent medical evaluation. Medical history interview, basic audiological assessment (tonal and vocal audiometry and acoustic immittance measurements), behavioral assessment of CAP and electrophysiological evaluation were performed in this sequence. The participants were referred by their assisting neuropediatricians.

The test battery chosen to evaluate CAP was composed of the following tests: SSI (Synthetic Sentence Identification) or PSI (Pediatric Speech Intelligibility), DD (Dichotic Digits test), DCV (Dichotic Consonant-Vowel test), GIN (Gaps in Noise), PPS (Pitch Pattern Sequence) and MLD (Masking Level Difference), in accordance with the recommendations of the Brazilian Academy of Audiology (2016).^24^ The behavioral tests were performed in a soundproof booth, using a Harp audiometer (Inventis), connected to a notebook containing the behavioral test tracks.

The Dichotic Digits test (DD) was performed at 50 dBSL (decibel sensation level), with binaural presentation for the binaural integration and binaural separation steps. The Dichotic Consonant-Vowel test (DCV) was performed at 55 dBSL. The Synthetic Sentence Identification (SSI) and the Pediatric Speech Intelligibility (PSI) tests were performed at 40 dBSL for the main message, with an ipsilateral competitive message presented in two conditions (0 and −15 dB). The Gaps in Noise (GIN), Pitch Pattern Sequence (PPS) and Masking Level Difference (MLD) tests were performed at 50 dBSL. Recording and analysis were performed according to the guidelines of the tests. Calculation of intensity of presentation (dBSL) was based on the three-tone average of the airway at the frequencies of 500, 1000 and 2000 Hz. All the tests performed were previously trained with the patients to ensure that they had understood the tasks.

The electrophysiological assessment consisted of the P300 and mismatch negativity (MMN) tests. These potentials were recorded with the participants seated in a comfortable chair. The skin was cleansed with gauze moistened with an antiseptic scrub solution. Electrodes were placed using EEG conductive paste and adhesive tape: the ground electrode was attached to the forehead and the active electrode (Fz), close to the scalp; an electrode (M1) was attached to the right mastoid and electrode (M2) on the left mastoid; finally, earphones (EarphoneTONE™ GOLD) were placed on both ears.

The assessment was only initiated when impedance was less than or equal to 5Ω (ohms). Electroencephalogram (EEG) scanning was performed to verify artifacts that might interfere with the test. A Brainstem Auditory Evoked Potentials (BAEP) scan was performed at 80 dBHL to check the integrity of the auditory pathway.

MMN was obtained monaurally, with frequency of 1000 Hz for the frequent stimulus and 2000 Hz for the rare one (50 cycles each), at an intensity of 70–80 dBHL for both stimuli, with a rate of 1.8 stimuli per second. A total of 150 stimuli were averaged, using the oddball paradigm (90 %/10 %) and alternating polarity. Data acquisition used 200 μV full scales, 1 Hz high-pass filter, 20 Hz low-pass filter, Notch-YES, time window of 500 ms, and trace amplitude up to 7.5 μV. To perform the MMN, the children watched a video (with volume off) on a tablet to distract their attention from the auditory stimuli.

For P300, the stimuli were binaural with tone burst and plateau of 20 ms and 5 m rise-fall times, at frequencies of 1000 Hz for the frequent stimulus (80 % of the presentations) and 2000 Hz for the rare one (20 % of the presentations), at 80 dBHL for both of them. With alternating polarity, the pace of stimulus presentation occurred at regular intervals of 0.8 pulses per second. During data acquisition, full scale was 200 μV, high pass filter 0.5 Hz, low pass filter 20 Hz, Notch-YES, and reading window was 1000 ms. The children were instructed to pay attention to the auditory stimuli presented, including the rare stimuli. The latency of the P300 was marked at the point of maximum amplitude of the wave.

Before the collection of electrophysiological data was started, all the subjects were informed about the execution steps of the tests so that they could understand the instructions correctly. MMN was performed prior to P300 to ensure completion of the requested task, since in MMN no attention should be paid to the stimulus, unlike in P300. To increase reliability, the electrophysiological records were analyzed by two evaluators at different times. Importantly, MMN was collected with monaural stimuli while P300 with binaural stimuli, because the same data collection parameters were maintained for the control group, and such collection had already been started. The Contronic® Masbe ATC Plus system was used. At least two samples were taken for each wave in order to check for replicability.

Quantitative variables were described through mean and standard deviation, while categorical variables, through absolute and relative frequencies. Student's *t*-test was used for comparison of means, and Pearson's Chi-square or Fisher's exact tests were applied for comparison of proportions. Student's *t*-test for paired samples was used for comparison between the right and left sides. Data were analyzed using the Statistical Package for Social Science (SPSS) 21.0 for Windows. For statistical decision criteria, the significance level of 5 % was adopted.

## Results

Thirty-four children and adolescents diagnosed with stroke at a reference outpatient clinic were invited to participate in the study. In view of the inclusion and exclusion criteria, 19 children and adolescents in the SG were included. The CG was composed of 19 children and adolescents with typical development, matched by age and sex. Table 1 shows the characteristics of the sample.

**Table 1:** Characterization of sample.

Variables	Study group (n = 19)	Control group (n = 19)
Age (years)	11.8 ± 2.6	11.8 ± 2.6
Age group – n (%)		
Children	6 (31.6)	6 (31.6)
Adolescents	13 (68.4)	13 (68.4)
Gender – n (%)		
Male	9 (47.4)	9 (47.4)
Female	10 (52.6)	10 (52.6)
Manual preference – n (%)		
Right-handed	14 (73.7)	18 (95)
Left-handed	5 (26.3)	1 (5.0)
Schooling (years)	5.6 ± 2.4	5.8 ± 2.1
Stroke type – n (%)		
Ischemic	15 (78.9)	
Hemorrhagic	2 (10.5)	
CVST	2 (10.5)	
Stroke side – n (%)		
RH	5 (26.3)	
LH	8 (42.1)	
Others	6 (31.6)	

CVST, Cerebral Venous Sinus Thrombosis; RH, Right Hemisphere; LH, Left Hemisphere.; *t*-test for independent samples; *p* <  0.05.

Table 2, Table 3 show the findings of the behavioral tests for CAP and the electrophysiological assessment of both groups.

**Table 2:** Comparison of behavioral tests between the study and control groups.

Variables	Study group (n = 19)	Control group (n = 19)	*p*
PSI/SSI 0			
RE	7.9 ± 1.5	9.4 ± 0.8	< 0.001
LE	7.8 ± 1.2	9.4 ± 0.8	< 0.001
PSI/SSI -15			
RE	5.8 ± 1.8	8.6 ± 1.4	< 0.001
LE	5.9 ± 1.5	8.7 ± 1.5	< 0.001
DD integration (%)			
RE	78.8 ± 16.4	97.9 ± 3.7	< 0.001
LE	83.2 ± 15.2	95.5 ± 6.2	0.005
DD separation (%)			
RE	79.2 ± 13.5	97.6 ± 4.1	< 0.001
LE	89.7 ± 11.2	95.8 ± 5.9	0.057
PPSh (%)	79.0 ± 9.2	94.1 ± 8.8	< 0.001
PPSl (%)	46.2 ± 15.1	74.5 ± 16.6	< 0.001
MLD	9.7 ± 3.5	11.2 ± 2.8	0.179
GIN	9.8 ± 1.9	6.0 ± 1.2	< 0.001

*t*-test for independent samples; *p* < 0.05.

RE, Right Ear; LE, Left Ear; PSI, Pediatric Speech Intelligibility; SSI, Synthetic Sentence Identification; DD, Dichotic Digit test; PPSh, Pitch Pattern Sequence-humming; PPSh, Pitch Pattern Sequence-labeling; MLD, Masking Level Difference; GIN, Gaps In Noise.

**Table 3:** Comparison of electrophysiological tests between the study and control groups.

Variables	Study group (n = 19)	Control group (n = 19)	*p*
Latency MMN (ms)			
RE	367.1 ± 74.4	194.9 ± 46.6	< 0.001
LE	360.2 ± 70.9	186.1 ± 45.0	< 0.001
Amplitude MMN (μV)			
RE	6.5 ± 1.4	6.8 ± 3.9	0.803
LE	7.0 ± 3.2	6.3 ± 4.5	0.640
Latency P300 (ms)			
RE	484.3 ± 138.0	301.0 ± 8.9	< 0.001
LE	486.1 ± 135.4	305.0 ± 10.6	< 0.001
Amplitude P300 (μV)			
RE	13.6 ± 7.4	15.1 ± 3.6	0.582
LE	14.0 ± 6.8	14.4 ± 3.2	0.597

*t-*test for independent samples; *p* <  0.05.

RE, Right Ear; LE, Left Ear; MMN, Mismatch Negativity; ms, milliseconds; μV, microvolts.

Table 4 shows the comparisons of the behavioral and electrophysiological findings among the children and adolescents of the SG.

**Table 4:** Comparison of tests among age groups in the SG.

Variables	Children (n = 6)	Adolescents (n = 13)	*p*
PSI/SSI 0			
RE	7.8 ± 0.9	7.6 ± 1.7	0.873
LE	7.7 ± 0.9	7.8 ± 1.3	0.947
PSI/SSI -15			
RE	5.5 ± 1.3	5.9 ± 2.1	0.730
LE	6.2 ± 1.3	5.8 ± 1.7	0.641
DD integration (%)			
RE	73.5 ± 20.3	81.0 ± 14.9	0.404
LE	76.5 ± 10.5	86.0 ± 16.4	0.252
DD separation (%)			
RE	85.5 ± 12.0	76.7 ± 13.7	0.231
LE	90.0 ± 11.7	89.6 ± 11.6	0.947
PPSh (%)	75.5 ± 13.9	80.6 ± 7.3	0.462
PPSl (%)	43.3 ± 12.0	47.4 ± 17.0	0.722
MLD	8.0 ± 2.4	10.6 ± 3.8	0.189
GIN	11.0 ± 2.6	9.3 ± 1.3	0.096
MMN – latency (ms)			
RE	380.1 ± 33.3	360.6 ± 89.2	0.649
LE	375.8 ± 31.9	352.4 ± 84.7	0.566
MMN – amplitude (μV)			
RE	6.1 ± 2.5	6.8 ± 0.6	0.374
LE	6.2 ± 2.5	7.4 ± 3.5	0.511
P300 – latency (ms)			
RE	464.5 ± 130.2	496.6 ± 150.1	0.702
LE	469.6 ± 118.2	496.4 ± 152.0	0.745
P300 – amplitude (μV)			
RE	15.1 ± 9.0	12.6 ± 6.6	0.571
LE	15.0 ± 8.6	13.4 ± 6.1	0.697

*t*-Student test; *p* < 0.05.

RE, Right Ear; LE, Left Ear; PSI, Pediatric Speech Intelligibility; SSI, Synthetic Sentence Identification; DD, Dichotic Digit Test; PPSh, Pitch Pattern Sequence-humming; PPSh, Pitch Pattern Sequence-labeling; MLD, Masking Level Difference; GIN, Gaps In Noise; MMN, Mismatch Negativity; ms, milliseconds; μV, microvolts.

Because the sample of children and adolescents diagnosed with stroke and CVST was small, possible associations with type of stroke could not be investigated.

Table 5 shows the behavioral and electrophysiological findings according to the side of stroke lesion. Five participants diagnosed with a right-sided stroke and 8 participants with a left-sided stroke were included. In the comparison between locations, there was a significant difference only for amplitude of MMN in the LE.

**Table 5:** Comparison of tests between hemispheres affected.

Variables	Right hemisphere	Left hemisphere	*p*
PSI/SSI 0			
RE	7.8 ± 2.4	8.0 ± 0.7	0.865
LE	8.2 ± 1.1	8.2 ± 0.8	1.000
PSI/SSI -15			
RE	6.4 ± 2.6	6.2 ± 0.8	0.874
LE	6.4 ± 2.1	6.2 ± 0.8	0.849
DD integration (%)			
RE	87.5 ± 9.8	73.8 ± 21.2	0.217
LE	77.5 ± 18.7	90.0 ± 10.5	0.232
DD separation (%)			
RE	85.0 ± 9.4	72.9 ± 12.9	0.115
LE	83.0 ± 13.0	92.5 ± 8.2	0.174
PPSh (%)	80.5 ± 5.9	80.4 ± 7.9	0.983
PPSl (%)	41.7 ± 14.8	50.7 ± 16.9	0.476
MLD	9.2 ± 2.3	11.7 ± 4.6	0.308
GIN	8.8 ± 1.1	10.0 ± 1.8	0.207
MMN – latency (ms)			
RE	383.3 ± 61.4	339.2 ± 94.0	0.427
LE	367.5 ± 66.7	337.0 ± 89.4	0.571
MMN – amplitude (μV)			
RE	5.8 ± 1.2	6.6 ± 0.8	0.207
LE	3.7 ± 0.4	8.7 ± 3.2	0.013
P300 – latency (ms)			
RE	504.2 ± 96.3	497.0 ± 164.2	0.940
LE	499.8 ± 95.8	499.2 ± 167.3	0.995
P300 – amplitude (μV)			
RE	14.3 ± 7.2	13.7 ± 8.0	0.916
LE	14.8 ± 6.9	13.9 ± 6.9	0.853

Student's *t*-test for paired samples; *p* < 0.05.

RE, Right Ear; LE, Left Ear; PSI, Pediatric Speech Intelligibility; SSI, Synthetic Sentence Identification; DD, Dichotic Digit Test; PPSh, Pitch Pattern Sequence-humming; PPSh, Pitch Pattern Sequence-labeling; MLD, Masking Level Difference; GIN, Gaps In Noise; MMN, Mismatch Negativity; ms, milliseconds; μV, microvolts.

The findings of the DVC test were analyzed according to the side of stroke lesion. Responses were predominant in the Right Ear (RE) for 4 out of the 5 individuals with right-sided stroke, but in the Left Ear (LE) for 1 individual. Responses were predominant in the LE for 7 out of the 8 individuals with left-sided stroke, but in the RE for 1 individual.

## Discussion

Although there is a high prevalence as well as a high functional impact of impairments in CAP, deficits present in children and adolescents with stroke are still poorly explored.^25^, ^26^ In a recent study, Barros^27^ analyzed the audiological findings of children with a history of stroke and concluded that the patients did not present auditory impairments at the peripheral level, suggesting the need for CAP assessment.

According to the literature, neurological changes resulting from stroke vary according to the location and extent of the injury. In the present study, children and adolescents diagnosed with stroke – ischemic, hemorrhagic or cerebral venous sinus thrombosis – were evaluated. According to medical records, strokes were classified according to the level of the lesion in the central nervous system. Because of the variation in location and extension data, we chose to describe them only according to hemispheric lateralization – right or left hemisphere. Cases in which stroke was described at other level of CNS were classified as ‘Other’.

In the present study, CAP skills were investigated in children and adolescents with stroke through behavioral and electrophysiological tests.

The behavioral tests in use were selected to assess binaural integration and binaural separation, figure-ground, temporal resolution and ordering, and binaural interaction skills. In the analysis of the findings, the SG presented worse performance in all the tasks, when compared to the CG. The findings were significant for the PSI/SSI, GIN and PPS tests, indicating a loss in figure-ground, temporal resolution and ordering skills. For the DD test, there was a statistical difference for the binaural integration stage, indicating impairment in this ability. In the binaural separation step, there was no significant difference for LE. Although the SG presented worse performance in the MLD test, there was no significance, indicating less impairment in the ability of binaural interaction.

Although studies with children diagnosed with stroke are scarce in the literature, they have reported a moderate impairment of hearing abilities.^20^, ^21^ Damage in dichotic listening has been reported in tasks that require separation and integration of verbal and non-verbal auditory information.^20^, ^28^ In the present study, dichotic listening in binaural integration and separation tasks were assessed using the DD test. In the comparison between SG and CG, children and adolescents with stroke presented worse performance, corroborating findings in the literature.^20^, ^28^ There was no statistical difference only for the binaural separation step (left ear).

Because it is a verbal dichotic test, the dominant hemisphere for language (in general, the left hemisphere) is necessary for verbal perception and labeling of linguistic stimuli. Thus, a good performance in the RE requires integrity of the pathways that directly reach the Left Hemisphere (LH), while a good LE performance requires integrity of the paths that reach the Right Hemisphere (RH), as well as callosal efficiency and LH integrity.^29^ That is, in cases of unilateral lesion in the RH, lower hearing thresholds are expected in the LE, because in cases of unilateral lesion in the LH, lower thresholds are expected in either the RE or in both ears. In cases of unilateral stroke, this pattern was found in the present study, with findings of lower thresholds in the ear contralateral to the lesion. These findings are in line with those of previous studies.^2^, ^20^, ^21^, ^30^

Murphy et al.,^30^ when analyzing the findings of the DD in a child with a history of unilateral stroke in the LH, found 100 % performance in the LE and 0 % in the RE. The authors suggested that these findings may indicate reorganization and language development in the RH as a result of strong plasticity usually following early brain injury.

In the DCV test, asymmetric responses are expected, with predominance of responses to one of the ears. In general, the LH is the dominant hemisphere for language, and therefore, there tend to be more responses for the syllables heard in the RE.^29^ In cases of neurological lesions, this asymmetry may change.^20^ In the present study, the findings were analyzed according to the side of stroke lesion. There was a pattern of responses: when the lesion occurred in the RH, responses were predominant in the RE, and when the lesion was present in the LH, the LE had a greater performance.

In a recent study, when using the DCV for assessment of children with stroke, the authors also found a tendency for more responses in the RE when the lesion was in the RH. However, in children with LH lesions, responses were more heterogeneous. The authors refer to these findings as a reorganization of language after the vascular event, and they may be associated with multiple factors, including location, extent and type of injury.^20^

According to the literature, children with stroke also have significant deficits in temporal processing.^21^ Temporal processing is critical for the wide range of daily auditory tasks such as discrimination of subtle signals and discrimination of similar words. It can be subdivided into two skills: temporal ordering and temporal resolution, evaluated in this study by the PPS and GIN tests, respectively.^2^

In the present study, there was a statistical difference between the groups for PPS, in both tasks, humming and labeling (*p* < 0.001). This difference indicates a deficit in temporal ordering ability. Temporal ordering is based on the principle that normal-hearing subjects are able to perceive, associate and interpret the non-verbal patterns of the message received, e.g., rhythm, intonation and melody. Because the PPS is a complex test, a good performance requires integrity of both cerebral hemispheres. As the humming stage does not require a verbal task, the distinction between tones is mediated by the non-dominant hemisphere for language (usually the RH). The labeling task, in turn, depends not only on the RH to perform the analysis and recognition of the acoustic contour, but also on callosal efficiency and the LH, for verbalization of the tonal pattern.^29^, ^31^

In a recent study,^30^ the authors found normal performance in PPS when evaluating a child with history of stroke in the LH. Their results are different from those found in the present study.

In the analysis of GIN, the SG had a significantly worse performance than the CG (*p* <  0.001), indicating a deficit in the temporal resolution ability. Changes in this ability could cause difficulty in identifying small acoustic variations of speech and difficulty in producing sounds correctly or in interpreting the message heard.^32^

According to the literature, findings of low performance in behavioral assessments of CAP indicate impaired auditory functions, which can impact the performance of several daily life activities, with implications for learning and socialization.^21^ Functional limitations can be observed in people’s difficulty in following oral instructions and understanding speech, even in the absence of peripheral auditory impairment.^30^

As the combination of objective and subjective methods has increased the accuracy of audiological diagnosis, the electrophysiological assessments of MMN and P300 were included in the present study. Electrophysiological measurements have been used to assess the efficiency of behavioral measurements as well as to check the functional and structural integrity of the neural components of the auditory pathway.^1^, ^7^, ^33^

Because of its cognitive demand, the P300 is crucial to capture cognition-related potentials generated in the CANS. It essentially reflects the activity of cortical auditory areas responsible for the abilities of attention, discrimination, integration and memory.^34^ MMN, in turn, reflects the pre-attentive discrimination and auditory memory of the subjects evaluated, and it also refers to processing and involuntary attention abilities.^35^, ^36^, ^37^

P300 and MMN are analyzed for latency, which represents the speed of information processing, and amplitude, which seems to be directly related to the amount of neuronal structure involved in responses. However, since it is a highly variable parameter, few studies to date have included an analysis of them.^7^, ^36^, ^38^, ^39^ Findings of increased latency or decreased amplitude may indicate a decline in neuronal activation, and they are considered to be objective indicators of clinical and subclinical changes.^40^, ^41^, ^42^

In the present study, children and adolescents with stroke presented significantly slower latency results for MMN and P300 when compared to the GC. This is indicative of possible deficits in the abilities assessed by these potentials. As for amplitude, there was no statistical difference between the amplitude values of the SG and the CG.

Because of the complexity of the areas involved, it is known that P300 is important to provide further insights into neural processes. It can be used to measure and monitor neurophysiological changes to SNAC, especially in cases of auditory processing deficit.^43^ However, no other studies were found to include this assessment for the post-stroke pediatric population.

The latency and amplitude values of MMN found in the present study agree with those from the literature. In a recent study, the MMN findings of 18 children and adolescents diagnosed with stroke were analyzed. When compared to a control group, higher latency values were found for the study group. There were statistical differences, indicating that discrimination skills, involuntary attention and sensorial memory may be impaired. No statistical differences were found for amplitude between the groups.^44^

Other studies that included electrophysiological assessment in this population were not found in the literature.

As a result of sample size, the association between the behavioral and electrophysiological findings and type of stroke could not be investigated. In addition to sample size, other limitations of the present study include the heterogeneity of stroke characteristics and their clinical presentation. For these reasons, no associations were found between the behavioral and electrophysiological findings and the different age groups of the study, nor was there any association of the present findings with the side of stroke lesion as a variable, except for amplitude of MMN in the LE, possibly because of high variability, as mentioned above.

There were few studies in the literature that included the assessment of CAP in children and adolescents diagnosed with stroke, either through behavioral or electrophysiological tests. Thus, the present study may offer relevant data for future studies in this population.

## Conclusion

It can be concluded that children and adolescents diagnosed with stroke present a worse performance in the electrophysiological and behavioral assessments of CAP when compared to a control group.

## Funding

CAPES (Coordenação de pessoal de nível superior), UFRGS (Universidade Federal do Rio Grande do Sul).

## Conflicts of interest

The authors declare no conflicts of interest.
